# Long and Short Duration Exposures to the Selective Serotonin Reuptake Inhibitors (SSRIs) Fluoxetine, Paroxetine and Sertraline at Environmentally Relevant Concentrations Lead to Adverse Effects on Zebrafish Behaviour and Reproduction

**DOI:** 10.3390/toxics11020151

**Published:** 2023-02-04

**Authors:** Ananda Baskaran Venkatachalam, Bailey Levesque, John C. Achenbach, Jane J. Pappas, Lee D. Ellis

**Affiliations:** 1 National Research Council, Halifax, NS B3H 3Z1, Canada; 2 New Substances Assessment and Control Bureau, Health Canada, Ottawa, ON K1A 0K9, Canada

**Keywords:** zebrafish, SSRIs, environment, stress, anxiety, fecundity, fertility

## Abstract

Selective serotonin reuptake inhibitors (SSRIs) are currently the most prescribed class of psychotropic medications. Their increased global manufacture and use have become growing concerns for aquatic toxicologists and environmental biologists, who assess both the direct and indirect effects of substances on the environment and on human health. In order to assess the potential impact of environmentally relevant levels of SSRIs on fish development, behaviour and reproduction, we exposed juvenile and adult zebrafish to a select group of SSRIs using two separate exposure paradigms. In the first paradigm, juvenile zebrafish were exposed to Fluoxetine (Prozac), Paroxetine (Paxil), Sertraline (Zoloft) or a mixture of the three beginning at environmentally relevant levels (10 µg/L) for 135 days (long-term exposure) beginning at 5 days post fertilization (dpf). In the second paradigm, adult zebrafish were exposed to matching concentrations of the same SSRIs for 35 days (short-term exposure). The long-term exposure paradigm proved to have little to no overt effect on growth or development at sub-lethal concentrations (10 and 100 µg/L). However, both the stress/anxiety response (novel tank tests) and reproduction (fecundity and fertility) were dramatically reduced. Importantly, the short-term exposure of reproductively mature fish led to similar adverse effects on both the stress response and reproduction. Following both the short and long duration exposure paradigms, a 2-week washout period led to a small reduction in the adverse effects. These findings highlight the potential for SSRIs to negatively impact population dynamics in zebrafish and may be of particular value should they be found in other fish species in the environment.

## 1. Introduction

Globally, the use of antidepressants has risen sharply since the beginning of the COVID-19 outbreak and is projected to continue to rise [1]. This is thought to have led to increased levels of antidepressants in the environment. This may be particularly important for the class of antidepressants known as selective serotonin reuptake inhibitors (SSRIs), which are frequently used to treat a wide assortment of conditions including major depressive disorder, obsessive–compulsive disorder, post-traumatic stress disorder, social anxiety disorder and panic disorder. SSRIs have already been reported at high levels in effluents and are found in surface water globally, due, in part, to the difficulty in removing them from wastewater [2,3]. Importantly, tests of surface and wastewater samples from across Canada have revealed the presence of a number of SSRIs, namely, Citalopram, Escitalopram, Fluoxetine, Fluvoxamine, Paroxetine and Sertraline which can be detected at levels between 2 and 500 ng/L [4,5,6,7,8,9]. In addition, a recent study has reported paroxetine at 3.4 ug/L and sertraline at 5.1 µg/L in wastewater treatment plant (WWTP) effluents from manufacturing sites across Canada [10]. Given the number of SSRIs that can be detected in wastewater along with the levels that have been measured, when SSRIs are released together in the same effluent, their total concentration may well exceed the 8.5 µg/L level based on the sum of paroxetine and sertraline that was reported by Kleywegt et al., 2019 [10]. As such, the potential for them to have a cumulative effect is now becoming a point of interest as the number of SSRI prescriptions increases [11]. 

SSRIs have been shown to have a wide array of adverse effects on many different aquatic organisms. For example, in algae, growth was found to be inhibited at concentrations as low as 18.6 µg/L [12] in the snail, righting time was found to be increased at concentrations as low as 34.2 µg/L [13] in the Japanese medaka, low level abnormalities in offspring were found to be increased at concentrations as low as 0.1 µg/L [14]; and in the zebrafish, the mortality rate was found to be increased at concentrations as low as 0.01 µg/L [15] Importantly, fish are reportedly attracted to WWTPs and their outfalls and are therefore exposed to the higher concentrations of contaminants, including SSRIs, found therein [16,17].

A number of studies have been conducted using zebrafish as a model organism to test the effects of SSRI exposure on fish and potentially shed light on the effects on other vertebrates such as humans, given their high genomic and disease ortholog similarity [18] In zebrafish, it has been shown that exposure to SSRIs can affect swimming activity [19,20,21,22,23,24], the visual motor response [25], and reproductive functions [26,27,28]. In Japanese medaka, mating behaviour was shown to be affected by SSRIs [29]. The exposure paradigms for the studies tend to vary, with larval, juvenile and adult fish exposed from short (hours to days) to long (months) durations. 

In the current study, we used juvenile and adult zebrafish to assess the effects of short-term (35 day) and long-term (135 day) exposures to environmentally relevant levels of a select group of SSRIs on toxicity, growth, behavior and reproduction. In addition, we tested the combined effects of the SSRIs, as we hypothesized that the mixtures would lead to effects that differ from single SSRI exposures. We selected three SSRIs that are detected globally in wastewater and surface water, namely, Fluoxetine, Paroxetine and Sertraline [3]. It was found that exposures to the SSRIs at environmentally relevant levels had nominal effects on growth but had important effects on the behavioural stress response and on fecundity and fertility. In addition, SSRI mixtures led to similar effects to the single compounds under certain conditions. These results suggest that environmentally relevant concentrations of SSRIs may have effects on fish populations in general and that it is the cumulative concentrations of SSRIs and their effects that may need to be monitored at this time.

## 2. Materials and Methods

### 2.1. Chemicals

Fluoxetine hydrochloride (CAS# 56296-78-7), Paroxetine hydrochloride hemihydrate (CAS# 110429-35-1), Sertraline hydrochloride (CAS# 79559-97-0), dimethyl sulfoxide (CAS# 67-68-5), tricaine methanesulfonate (CAS# 886-86-2), and water (sterile filtered, Bioreagent grade) were purchased from Sigma-Aldrich Canada (Oakville, ON, Canada). Working stocks of Fluoxetine hydrochloride and Paroxetine hydrochloride hemihydrate were made by dissolving the powders in water at 10 and 5 mg/mL, respectively. Sertraline hydrochloride was dissolved in dimethyl sulfoxide (DMSO) at 10 mg/mL. These stocks were stored at 4°C. Methanol used in SSRI extractions was distilled-in-glass grade (Caledon Laboratories Ltd., Georgetown, ON, Canada). Mobile phases used in the liquid chromatography–high resolution mass spectrometry (LC-HRMS) were prepared with deionized water obtained from a Milli-Q system and LC-MS grade acetonitrile and LC-MS grade formic acid (Fisher Chemical, Ottawa, ON, Canada).

### 2.2. Stability of SSRIs

In order to assess the stability of the SSRIs over the 5-day exposure period chosen for the static exposure water tank renewal period, a nominal concentration of 1.5 ng/mL of each SSRI was monitored over the 5-day period (*n* = 3 tanks per SSRI). The 20 mL samples were removed from 3 L tanks daily and the SSRIs were extracted using Oasis HLB solid phase extraction columns (Waters Canada, Mississauga, ON, Canada) following the manufacturer’s instructions. SSRIs were eluted from the columns with 100% methanol and eluates were analyzed on an ACQUITY UPLC BEHC18 column (2.1 × 100 mm, 1.7 µm) using an UltiMate^TM^ 3000 LC pump coupled to an Exactive™ mass spectrometer (Thermo Fisher Scientific, Waltham, MA, USA). The limit of quantitation (LOQ) was found to be 0.1 ng/mL. Mixtures were not tested for SSRI stability since loss (which is either due to degradation, or adsorption to tank material, or both) is assumed to be independent of the presence of other SSRIs. It was found that all three of the compounds were stable over the exposure period with the maximum reduction of 20% found for paroxetine at day 5 (Supplemental Scheme S1).

### 2.3. Experimental Animals: Husbandry

All zebrafish (*Danio rerio*) larvae used were obtained by breeding adult wild-type AB/Tub hybrids, obtained from the breeding colony at the National Research Council’s zebrafish facility. For long-term exposures, age-matched embryos were sorted for fertilization at roughly 4 h post fertilization (hpf) and kept in E3 media (5 mM NaCl, 0.17 mM KCl, 0.33 mM CaCl_2_-2H_2_O, 0.33 mM MgSO_4_-7H_2_O) in 10 × 150 mm disposable polystyrene Petri dishes at 28.5 °C until used in the exposure assay at 5 days post fertilization (dpf). For short-term exposure experiments, adult AB/Tub hybrid zebrafish were obtained from the facility mentioned above. All adult zebrafish husbandry and breeding were carried out in accordance with the Canadian Council of Animal Care (CCAC) guidelines.

At 5 dpf, fry were transferred into a static-water fry rearing system (mesh-bottom baskets held within 3 L polycarbonate tanks) (Pentair Aquatic Ecosystem, Apopka, FL, USA). Water sourced from the main recirculating aquaria system at the National Research Council’s Zebrafish facility was renewed manually every 5 days. Fish densities were maintained at 25 fry (5–30 dpf) per basket with 2 baskets per tank containing 2.4 L of water. At 30 dpf, baskets were removed, and fish were allowed to swim freely directly in the tanks at a density of 15 fish per tank in 2.4 L of water. Room and water temperature ranged from 26 to 28 °C, and fish were kept at a photoperiod of 14 h light:10 h dark. Fish aged 5 to 60 dpf were fed 200 µL of a slurry per basket or tank twice a day made freshly by suspending 12.5 mg of Gemma Micro 75 (from 5–30 dpf) or Gemma Micro 150 (from 30–60 dpf) per mL of system water; fish older than 60 dpf were fed 10 mg of Gemma Micro 300 per tank twice a day (Skretting Canada, Vancouver, BC, Canada).

### 2.4. Experimental Design: Exposure Periods

***Long-term exposures.*** For long-term exposure experiments, 5 dpf juvenile zebrafish were exposed to Fluoxetine, Paroxetine, or Sertraline at 10, 100 or 200 μg/L, based on previously published work [21,29,30,31,32,33], for a total of 135 days. 

Mixtures of all three SSRIs at equal concentrations that, when combined, added up to 10, 100, or 200 µg/L were also tested. Since Sertraline working stocks were made in DMSO, the resulting 10, 100, and 200 µg/L Sertraline exposures contained negligible concentrations of DMSO (0.0001, 0.001, and 0.002 % (*v*/*v*), respectively) and the resulting 10, 100, and 200 µg/L SSRI mixture exposures also contained negligible concentrations of DMSO (0.000033, 0.0003, and 0.0006% (*v*/*v*), respectively). 

**Figure 1:**
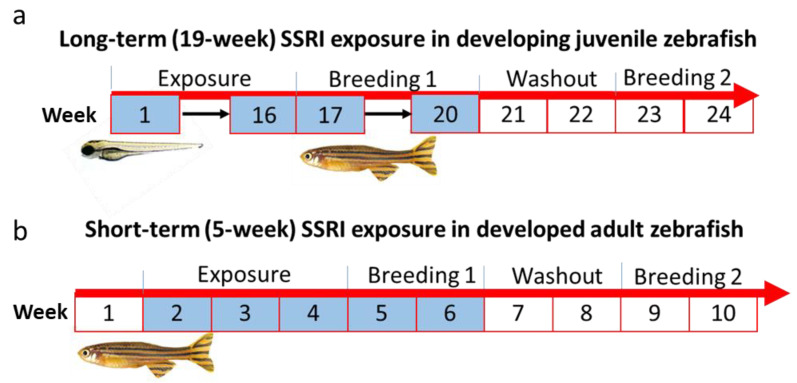
Treatment paradigms. (**a**) Long-term exposure testing from 5 to 170 days post fertilization (dpf). (**b**) Short-term exposure testing 63 day testing paradigm. Blue boxes represent treatment periods.

Each tank contained up to 50 fish from 5–30 dpf or up to 15 fish from 30–170 dpf. There were three replicate tanks for each exposure group along with a separate tank for controls. Controls were not exposed to SSRIs but were housed in otherwise identical conditions. Water was renewed every 5 days with working stocks of SSRIs pipetted directly into a new tank containing 2 L of clean water. An additional 400 mL of clean water was then added to the tank to ensure proper mixing of the drug. Fish were then transferred by netting to a new exposure tank. Exposures were followed by a 2-week washout period before a second breeding assessment was carried out for another 2 weeks (Figure 1a). Fish were euthanized at the conclusion of the study. 

***Short-term exposures.*** For short-term experiments, adult fish (10–14 months old) were exposed to the same SSRIs as for the long-term experiments or their combination at 10 μg/L for 3 weeks and breeding was assessed for 2 weeks (35 day exposure). This was followed by a 2-week washout period before a second breeding assessment was carried out for another 2 weeks (Figure 1b). Each exposure group had three replicate tanks along with a control (untreated) group; each tank contained 20 fish. Fish were euthanized at the conclusion of the study.

### 2.5. Morphological Data Collection and Survival

At 30 and 60 dpf, fish from each tank were photographed and Nikon Elements software was used to measure the standard length of each individual fish. At 90 and 120 dpf, an anaesthetic dose of tricaine methanesulfonate (168 µg/mL) was used during sampling, and immediately following anaesthesia, fish were weighed and photographed, and length was again determined using Nikon Elements software. 

### 2.6. Novel Tank Test (NTT) 

At 90 and 120 dpf, potential anxiolytic effects were assessed using a standard novel tank test (NTT) based on previous literature using the novel tank diving paradigm [34] Following placement in a novel tank, zebrafish generally dive to the bottom to seek protection and remain there presumably as long as they are enacting predator evasion behaviour. Therefore, we quantified the fraction of time fish spent in the bottom third of the water column as a measure of anxiety. In our experiment, individual fish were removed from their home tank, placed in a 3 L tank and immediately video recorded for 6 min. Noldus EthoVision XT software (version 15.0.1418) was used to track fish movement, to quantify the amount of time spent in the bottom third of the tank and to measure the total distance travelled during the trial. Twenty fish were assessed per exposure group including the control groups.

### 2.7. Reproductive Success

***Long-term exposures.*** As zebrafish generally reach sexual maturity by 120 dpf, the experiments designed to assess reproductive success were performed between 121 and 170 dpf. There were two breeding tests for each individual conducted over a 3 week period. Each fish was bred a single time during the first week, followed by a 1 week rest period and a subsequent second breeding test during week 3.

One day prior to trials, single mating pairs were placed in breeding baskets within tanks such that the male and the female were separated by a clear plastic divider. The dividers were removed at 8:30 AM when the lights in the facility automatically came on. The fish were left to spawn for 2 h and then placed back in holding tanks, as described earlier. Eggs were collected and placed in a 28.5 °C incubator. Roughly 3–5 h later, fertilized/total egg counts were performed, and fertilized eggs were returned to the incubator at a density of 25 eggs per Petri dish (~25 mL media). The next morning, normally developing, viable eggs were counted using a Leica MZ6 modular stereomicroscope. 

Clutch size and fertilization rate were chosen as indicators of reproductive success. Clutch size refers to the number of eggs released in a single spawning event and may be a plastic trait associated with female and/or male mate quality [35]. Individual clutches were counted and averaged, including unsuccessful spawning events as 0. Unfertilized eggs were counted at 6 hpf and 24 hpf. The number of fertilized and unfertilized eggs in each clutch was used to calculate the percent fertilization. Fertilization rate was used as a proxy for male mate quality [35]. 

***Short-term exposures.*** Short-term SSRI exposures were conducted in order to determine whether or not they lead to observable effects and to determine if the effects are similar to the long-term exposure effects. Adult fish (10–14 months old) were exposed to the SSRIs for 3 weeks. Fecundity was assessed with the protocol used for the long-term exposed fish for 2 weeks. Fish were re-assessed following a 2-week washout period followed by a 2-week re-breeding period (see Figure 1). As for the long-term exposures, this last step was performed using the same fish, though different mating pairs were assessed.

### 2.8. Statistical Analyses

Generalized linear models were used to assess the effects of drug exposure on growth, survival, sex ratio and reproductive parameters. Appropriate distributions were used to fit each model, and statistical significance with respect to controls was determined using one-way ANOVAs followed by Dunnett’s multiple comparisons tests. GraphPad PRISM^®^ (version 5) (San Diego, CA, USA) was used for these analyses. Data are expressed as means ± standard deviation (SD). Significance was set at *p* < 0.05. 

## 3. Results

### 3.1. Growth, Behaviour and Survival

**Figure 2:**
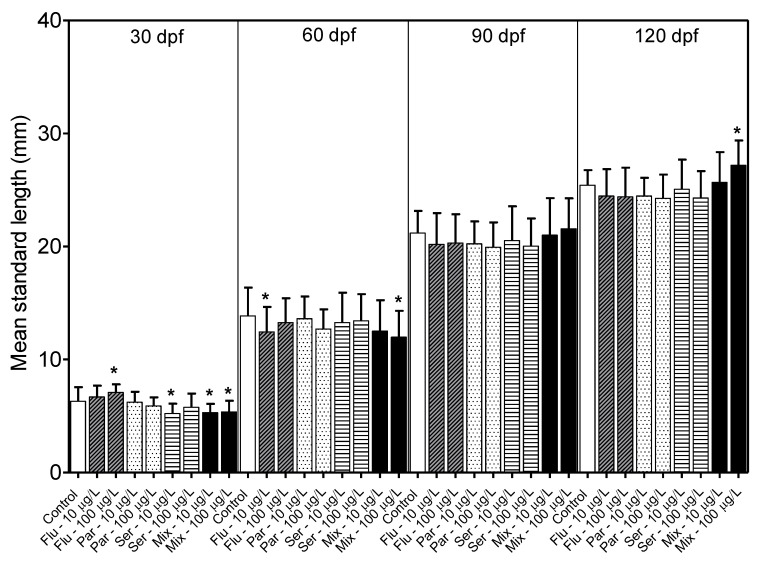
Mean standard length (mm) of fish measured at 30, 60, 90 and 120 days post fertilization (dpf). Flu—Fluoxetine; Par—Paroxetine; Ser—Sertraline; Mix—Mixture. Statistical significance with respect to the control fish group was determined using a one-way ANOVA followed by a Dunnett’s multiple comparisons test (* *p* < 0.05).

**Figure 3:**
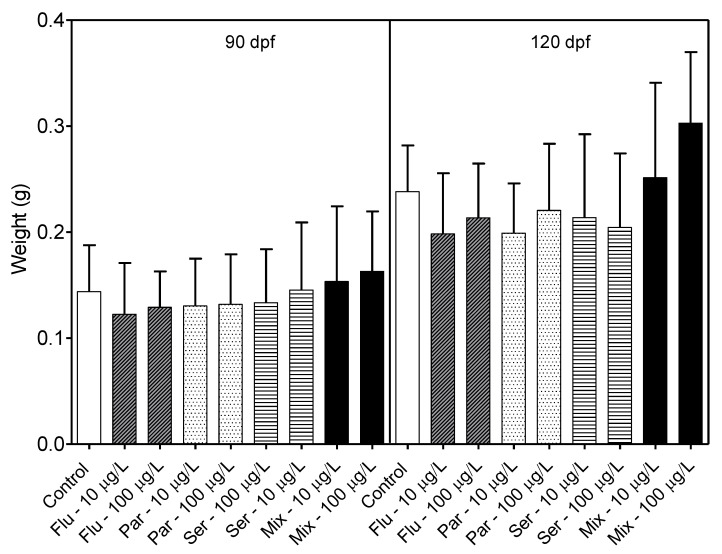
Weight of fish (g) measured at 90 and 120 days post fertilization (dpf). (Flu—Fluoxetine; Par—Paroxetine; Ser—Sertraline; Mix—Mixture). Statistical significance with respect to the control fish group was determined using a one-way ANOVA followed by a Dunnett’s multiple comparisons test.

By 30 dpf, exposure to 200 μg/L of the SSRIs alone or in combination exhibited a low survival rate of < 6% and this concentration group was removed from the study. Exposures to 10 and 100 μg/L of the SSRIs did not lead to statistically significant differences in survival rates compared to controls. Fish growth was assayed by measuring individual fish length at 30, 60, 90 and 120 dpf and individual fish weight at 90 and 120 dpf and averaging per group. Generalized linear models fitted to a normal distribution were used to examine the effects of drug exposure on length, weight and survival. Analyses revealed a nominal effect of SSRI exposure on length at 30 and 60 dpf, but no effect on length or weight at 90 and 120 dpf (Figure 2 and Figure 3).

### 3.2. Novel Tank Test (NTT)

**Figure 4:**
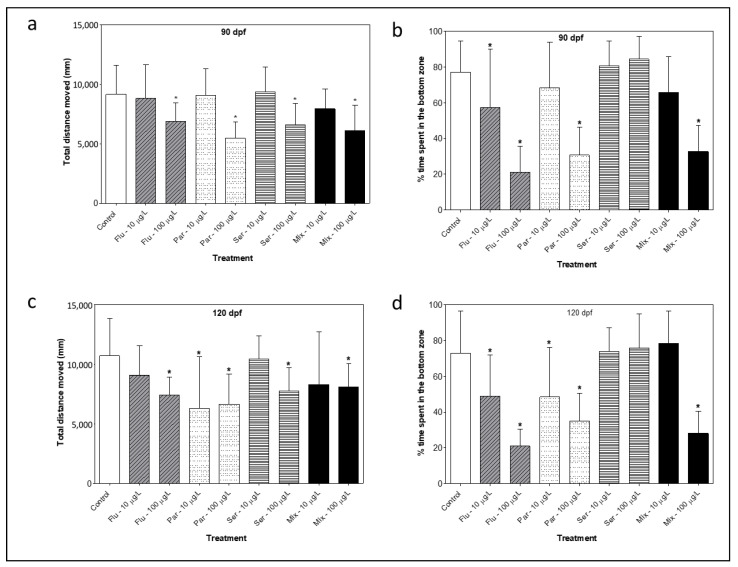
At 90 and 120 days post fertilization (dpf)**,** total activity of fish during 6 min experiments quantified as the total distance moved (mm) (**a**,**c**) and % time spent by fish in bottom zone of the tank during 6 min experiments (**b**,**d**). (Flu—Fluoxetine, Par—Paroxetine, Ser—Sertraline, Mix—mixture of all three compounds). Statistical significance with respect to the control fish group was determined using a one-way ANOVA followed by a Dunnett’s multiple comparisons test (* *p* < 0.05).

The standard novel tank test (NTT) was used to assess the effects of SSRI exposure on stress/anxiety levels at 90 and 120 dpf (Figure 4). Upon entering a new environment, such as a novel tank, zebrafish attempt to seek safety by diving to the bottom of the tank [34] As such, the fraction of time spent at the bottom of the tank is considered a quantifiable measure of their stress/anxiety level. In this experiment, individual fish were removed from their tank, transferred to a new 3 L tank and recorded for 6 min to monitor their behavioural patterns. Ethovision XT software (Noldus) was used to track fish movement, to measure the time spent at the bottom third of the tank, and to determine the total distance travelled during the trial (*n* = 20 fish per exposure).

A statistically significant reduction in the total distance travelled was observed for all SSRI exposure groups at 100 μg/L (Figure 4a,c). This reduction was accompanied by a statistically significant reduction in the fraction of time spent at the bottom third of the tank for Fluoxetine, Paroxetine and the mixture at 100 μg/L (Figure 4b,d). In the 10 μg/L groups, a statistically significant reduction in the total distance travelled was observed for Paroxetine, accompanied by a statistically significant reduction in the fraction of time spent in the bottom third of the tank (Figure 4c,d). For Fluoxetine at 10 μg/L, there was a significant reduction in the fraction of time spent at the bottom of the tank (Figure 4d).

### 3.3. Fecundity and/or Fertility-Long-Term Exposures

**Figure 5:**
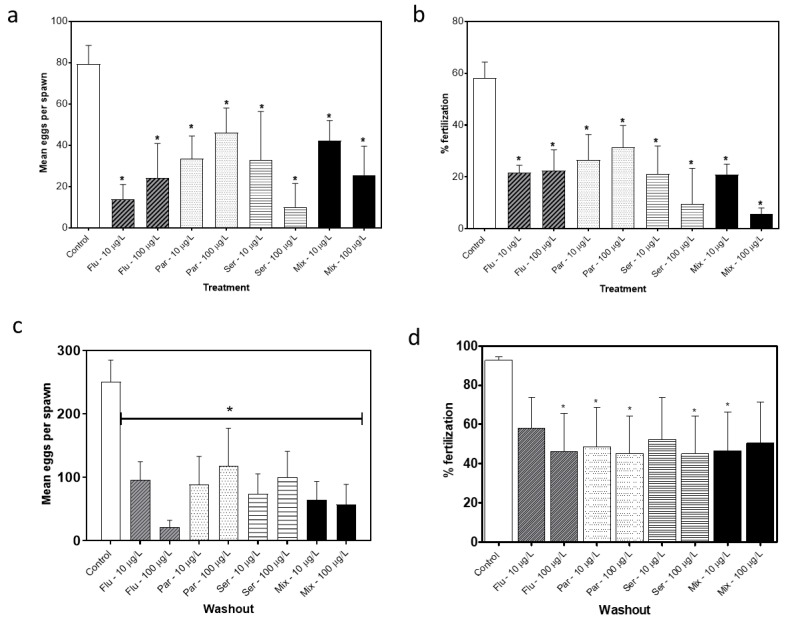
Mean number of eggs generated per single pair cross (all eggs regardless of viability) over a 3 week period for all fish in the treatment (Flu—Fluoxetine, Par—Paroxetine, Ser—Sertraline, Mix—mixture of all three compounds) and control groups (**a**) and following a 2 week washout period (**c**). Percent fertilization of clutches for the treatment (**b**) and washout groups (**d**). Statistical significance with respect to the control fish group was determined using a one-way ANOVA followed by a Dunnett’s multiple comparisons test (* *p* < 0.05).

Breeding was assayed between 120 and 170 dpf by comparing the fecundity and fertilization rates between exposure and control groups. Clutch size was used to assess fecundity, and fertilization rate was determined by counting the number of fertilized eggs in a clutch. Exposure to the SSRIs led to statistically significant reductions in both the clutch size and the number of fertilized eggs per clutch at both 10 and 100 µg/L for all of the SSRIs and the mixture compared to controls (Figure 5a,b).

In order to determine if the changes in fecundity and fertility were due to the direct effects of SSRI exposure or due, at least in part, to the longer-lasting effects of developmental exposure, a washout study was conducted. Following a 2-week washout period beginning at 140 dpf, the fish were again bred and re-assessed. Both the clutch sizes and the fertilization rates remained reduced for all of the SSRI exposed fish compared to the controls (Figure 5c,d). It should be noted that between the initial breeding period and the follow-up breeding after the washout, control clutch sizes increased from an average of 80 ± 9.02 to an average of 250 ± 75.96 eggs (Figure 5a,c), which, in our experience, is not unexpected since increasingly mature females generally have increasingly greater clutch sizes over this developmental timeframe.

### 3.4. Fecundity and/or Fertility (Short-Term Exposure)

**Figure 6:**
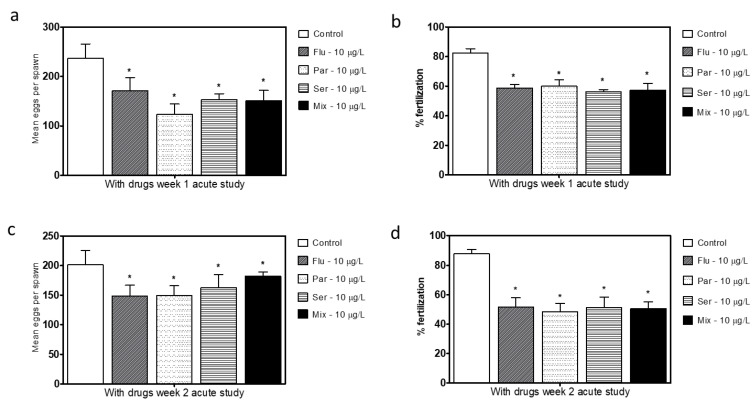
Mean number of eggs generated per single pair cross (all eggs regardless of viability) following the short-term exposure period during week 1 (**a**) and week 2 (**c**) for all treatment groups (Flu- Fluoxetine, Par—Paroxetine, Ser—Sertraline, Mix—mixture of all three compounds). Percent fertilization of clutches during week 1 (**b**) and week 2 (**d**). Statistical significance with respect to the control fish group was determined using a one-way ANOVA followed by a Dunnett’s multiple comparisons test (* *p* < 0.05).

**Figure 7:**
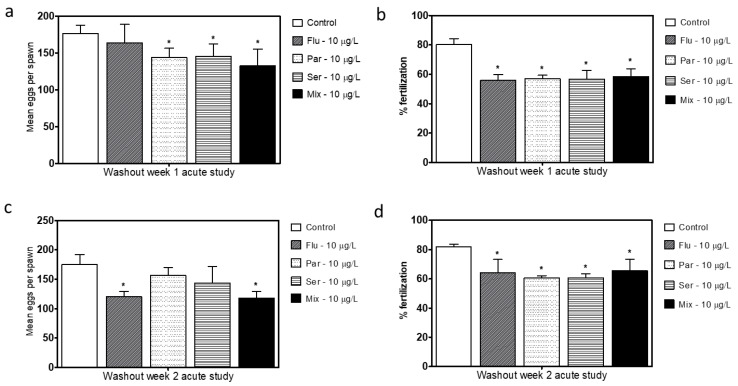
Mean number of eggs generated per single pair cross (all eggs regardless of viability) during week 1 (**a**) and week 2 (**c**) following the short-term exposure 2 week washout period for all treatment groups (Flu- Fluoxetine, Par—Paroxetine, Ser—Sertraline, Mix—mixture of all three compounds). Percent fertilization of clutches during week 1 (**b**) and week 2 (**d**) following the 2 week washout. Statistical significance with respect to the control fish group was determined using a one-way ANOVA followed by a Dunnett’s multiple comparisons test (* *p* < 0.05).

In order to further evaluate if the adverse effects of SSRIs on breeding in the long duration exposure testing were at least in part developmental in nature, a short-term exposure study was performed. Adult fish that were not previously exposed to SSRIs during development were exposed to the SSRIs or the equal part mixture at 10 μg/L for 3 weeks and breeding was conducted for 2 weeks (Figure 1). All exposure groups exhibited similar reductions in both clutch sizes and fertilization rates (Figure 6). The effects of the SSRI exposure were consistent for both of the test weeks, which suggests that the 3 week exposure period before the fertility testing is sufficient to reach the maximum effect that will be generated. Following the 2-week washout period when these parameters were re-assessed, in a manner similar to the long-term washout groups, the short-term washout groups still showed smaller clutch sizes and lower fertilization rates for the SSRI groups than the unexposed controls (Figure 7).

## 4. Discussion

In the long-term experiments, growth and survival rates were measured at 30, 60, 90 and 120 dpf. In the groups exposed to 200 μg/L SSRI, larvae were found to have low survival rates, which aligns with previously reported acute toxicity levels for Citalopram [36], Fluoxetine [26], and the tricyclic antidepressant Amitriptyline [37]. In the groups exposed to 10 μg/L SSRI, which is a concentration closer to environmentally relevant levels, and in the groups exposed to 100 μg/L SSRI, no statistically significant differences in survival rates, embryonic/larval morphologies or growth rates by 90 and 120 dpf for the substances tested were found. This parallels similar findings reported by Hong et al., for embryos and larvae exposed to <30 μg/L Vortioxetine [38], a serotonin modulator.

The novel tank test (NTT) [39] was used to assess stress/anxiety levels at 90 and 120 dpf in fish exposed to SSRIs for 12 weeks during development. A significant decrease in the fraction of time spent in the bottom zone of the tank was observed following exposure to fluoxetine and paroxetine at 100 μg/L. These findings are consistent with previous studies that have shown that early developmental exposures to SSRIs lead to the induction of behavioral abnormalities in fish [19,31,40,41] and in mammals [19,31,40,41]. In particular, it was previously shown that exposure of 3- to 5-month old zebrafish to Fluoxetine for 2 weeks led to a lower latency to enter the upper half of the tank, more time spent at the top, and more transitions to the top [34] It was also previously shown that 7-day exposures to the serotonin modulator Vorioxetine also led to an increase in the time spent in the upper half of the tank, accompanied by a shorter distance travelled and a lower average swimming velocity in the fish [38]. The normal behavioral response for the NTT, which is a preference for the bottom of the tank, is considered a stress response linked to predator avoidance. It is generally accepted that a reduction in this activity in a laboratory setting may reflect a reduced level of stress/predator avoidance in the wild, which ultimately may lead to diminished fish survival.

Similar to the current study, previous work has shown that SSRI exposure leads to adverse effects on reproduction in zebrafish. Citalopram exposure at 1, 10 and 100 μg/L led to 28%, 56% and 34% reductions, respectively, in the number of eggs produced [27] and fluoxetine exposure at 32 μg/L led to a 20% reduction in fecundity in zebrafish [28]. In the current study, there was a significant reduction in the fecundity of the fish exposed to the SSRIs with the greatest reduction in egg production found for Sertraline at 10 μg/L (68%) and the lowest for Paroxetine at 100 μg/L (33%). The current study also evaluated the effects of substance exposure on fertilization rate and there was a reduction for all of the exposure groups. The reduced egg production paired with the lower fertilization rate indicates that SSRI exposure has negative effects on the reproductive success of both female and male fish.

In order to determine if the changes found were the result of developmental effects or due solely or partially to the immediate effects of the SSRIs, two additional tests were performed: a washout study and a short-term exposure study. Following the washout of the SSRIs for 2 weeks in the long-term (24-week) study, the egg production and fertility partially recovered but did not reach control levels (Figure 5c,d). Following the short term (3–5 weeks) exposure of adults to the SSRIs there was a similar trend of decreased fecundity and fertility rates as those following the long-term exposure (Figure 6 and Figure 7); however, these reductions were not as large as those following the long-term exposures. Once again, following washout, the short-term exposure group had a partial recovery of both the egg production levels and fertilization rate, but the levels did not reach control levels. The results from both of the washout tests suggest that either fundamental changes were incurred or that the washout period was not long enough to completely eliminate the SSRI in the fish. Together, the results from the two exposure paradigms show the complexity of the SSRI effects, as the short-term exposure appears to produce a similar effect to chronic exposure and there is a prolonged effect of the SSRI exposure in the weeks following the 2-week washout periods.

The SSRI mixture groups included combined SSRI concentrations of each SSRI that, when added, matched the individual levels tested. As the results of the mixture testing groups are similar to those for the individual compounds, this suggests that, when combined at equimolar levels, the effects of the SSRIs may be additive. This allows the model to fulfill one of the primary concerns from an environmental risk assessment perspective which is to address the possibility that the SSRIs may have combined effects due to their similar mechanisms of action and that their combined levels in wastewater effluent and in surface water should be monitored. Future work will be required to match the ratios and levels of the SSRIs found in wastewater to evaluate and compare their individual and combined effects.

## 5. Conclusions

In summary, this study demonstrates that both short- and long-term exposures of zebrafish to SSRIs lead to pronounced adverse effects on behaviour and reproduction that may have negative effects on their overall reproductive success and survival rates in the wild. Importantly, the results of the short-term SSRI exposures may reflect the potential risks fish are subjected to as they travel through WWTP effluents. However, at this time the amounts of time different species of fish spend in these areas are not clear, which makes comparisons between the results of this study and the effects in the wild mostly speculative. The data from both the short- and long-term studies do suggest that while individual SSRIs may be found below effective concentrations (ECs) on their own, cumulative concentrations of multiple SSRIs may cross these EC boundaries and potentially cause detrimental effects that may be otherwise overlooked if single chemicals and pharmaceuticals such as SSRIs are only assessed one at a time. The next step in environmental risk assessment may then be to assess groups of chemicals having similar mechanisms of action together. Finally, given the complexity and similarities of the short- and long-term exposure findings, future work is required to tease apart the direct and indirect effects of SSRIs, in order to elucidate how development may be involved. Further studies using different SSRI combinations at different time-points within the zebrafish lifecycle may clarify the underlying mechanisms of these effects.

## Data Availability

Data available on request due to privacy restrictions.

## Supplementary Materials

The following supporting information can be downloaded at: https://www.mdpi.com/article/10.3390/toxics11020151/s1, Scheme S1: Stability of SSRIs under experimental conditions in the absence of zebrafish.
