# Supplementary material for: Long and Short Duration Exposures to the Selective Serotonin Reuptake Inhibitors (SSRIs) Fluoxetine, Paroxetine and Sertraline at Environmentally Relevant Concentrations Lead to Adverse Effects on Zebrafish Behaviour and Reproduction
Source: Toxics. 2023 Feb 4;11(2):151. doi: 10.3390/toxics11020151 (PMC9966831; doi:10.3390/toxics11020151)

toxics

Article

Long and Short Duration Exposures to the Selective Serotonin Reuptake Inhibitors (SSRIs) Fluoxetine, Paroxetine and Sertraline at Environmentally Relevant Concentrations Lead to Adverse Effects on Zebrafish Behaviour and Reproduction

Ananda Baskaran Venkatachalam [1], Bailey Levesque [1], John C. Achenbach [1], Jane J. Pappas [2] and Lee D Ellis [1,*]

Scheme S1. Stability of SSRIs under experimental conditions in the absence of larvae. The concentrations of SSRIs (nominal concentration 1.5 ng/mL) were monitored over the 5 day period chosen for static exposure tank water renewal (n=3 tanks for each SSRI). Levels detected each day are shown as a percentage of the starting concentration.

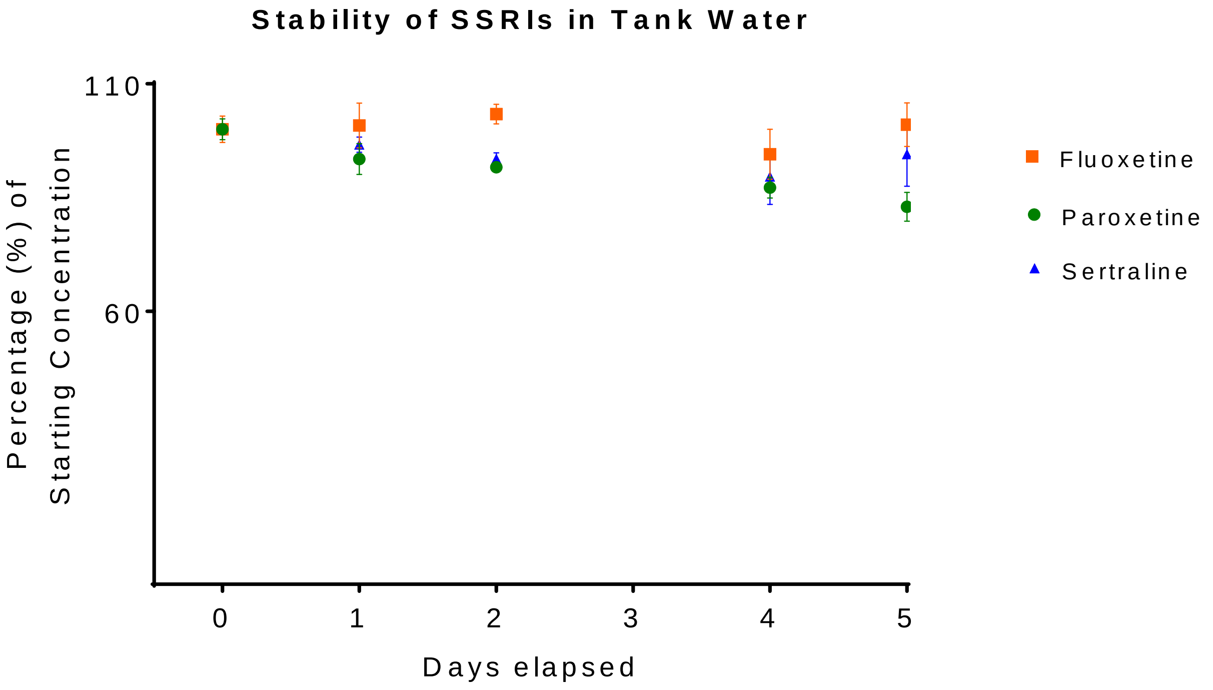

Supplement: Supplementary file 1 [file toxics-11-00151-s001.zip › toxics-2201863-supplementary.pdf]
